# Prediction Accuracies of Genomic Selection for Nine Commercially Important Traits in the Portuguese Oyster (*Crassostrea angulata*) Using DArT-Seq Technology

**DOI:** 10.3390/genes12020210

**Published:** 2021-02-01

**Authors:** Sang V. Vu, Cedric Gondro, Ngoc T. H. Nguyen, Arthur R. Gilmour, Rick Tearle, Wayne Knibb, Michael Dove, In Van Vu, Le Duy Khuong, Wayne O’Connor

**Affiliations:** 1 GeneCology Research Centre, University of the Sunshine Coast, 90 Sippy Downs Dr., Sippy Downs, QLD 4556, Australia; wayneknibb@gmail.com (W.K.);; 2 School of Science, Technology and Engineering, University of the Sunshine Coast, 90 Sippy Downs, QLD 4556, Australia; 3 Northern National Broodstock Centre for Mariculture, RIA1, Catba Islands, Hai Phong 180000, Vietnam; nguyenngocria1@gmail.com (N.T.H.N.); vuvanin@ria1.org (I.V.V.); 4 Department of Animal Science, College of Agriculture and Natural Resources, Michigan State University, East Lansing, MI 48824, USA; gondroce@msu.edu; 5 Statistical and ASReml Consultant, Orange, New South Wales 2800, Australia; arthur.gilmour@cargovale.com.au; 6 School of Animal and Veterinary Science, The University of Adelaide, Adelaide 5005, Australia; rick.tearle@adelaide.edu.au; 7 NSW Department of Primary Industries, Port Stephens Fisheries Institute, Taylors Beach, New South Wales 2316, Australia; michael.dove@dpi.nsw.gov.au; 8 Ha Long University, Uong Bi 200000, Quang Ninh, Vietnam; leduykhuong@daihochalong.edu.vn

**Keywords:** genomic selection, prediction accuracy, analysis methods, SNP marker density, genomic parameters

## Abstract

Genomic selection has been widely used in terrestrial animals but has had limited application in aquaculture due to relatively high genotyping costs. Genomic information has an important role in improving the prediction accuracy of breeding values, especially for traits that are difficult or expensive to measure. The purposes of this study were to ***(i)*** further evaluate the use of genomic information to improve prediction accuracies of breeding values from, ***(ii)*** compare different prediction methods (BayesA, BayesCπ and GBLUP) on prediction accuracies in our field data, and ***(iii)*** investigate the effects of different SNP marker densities on prediction accuracies of traits in the Portuguese oyster (*Crassostrea angulata*). The traits studied are all of economic importance and included morphometric traits (shell length, shell width, shell depth, shell weight), edibility traits (tenderness, taste, moisture content), and disease traits (*Polydora* sp. and *Marteilioides chungmuensis*). A total of 18,849 single nucleotide polymorphisms were obtained from genotyping by sequencing and used to estimate genetic parameters (heritability and genetic correlation) and the prediction accuracy of genomic selection for these traits. Multi-locus mixed model analysis indicated high estimates of heritability for edibility traits; 0.44 for moisture content, 0.59 for taste, and 0.72 for tenderness. The morphometric traits, shell length, shell width, shell depth and shell weight had estimated genomic heritabilities ranging from 0.28 to 0.55. The genomic heritabilities were relatively low for the disease related traits: *Polydora* sp. prevalence (0.11) and *M. chungmuensis* (0.10). Genomic correlations between whole weight and other morphometric traits were from moderate to high and positive (0.58–0.90). However, unfavourably positive genomic correlations were observed between whole weight and the disease traits (0.35–0.37). The genomic best linear unbiased prediction method (GBLUP) showed slightly higher accuracy for the traits studied (0.240–0.794) compared with both BayesA and BayesCπ methods but these differences were not significant. In addition, there is a large potential for using low-density SNP markers for genomic selection in this population at a number of 3000 SNPs. Therefore, there is the prospect to improve morphometric, edibility and disease related traits using genomic information in this species.

## 1. Introduction

The Portuguese oyster (*Crassostrea angulata*) is an important aquaculture species around the world, especially in Asian and European countries [1,2,3]. For example, the total Portuguese oyster production of China was around 1.35 × 10^6^ metric tonnes in 2014 [4], while the annual total production of Portuguese oyster in Vietnam was around 50,000 tonnes per year in 2018 [5]. Although the Portuguese oyster industry is developing rapidly, it is afflicted by disease and the sub-lethal effects of disease are economically damaging. There are two common parasites that can kill their host and otherwise dramatically reduce marketability. The parasite *Marteilioides chungmuensis* causes spots on the soft tissue of the Portuguese oyster; while polychaete “mud-worms” of the genus *Polydora* cause black blisters on the inner surfaces of the shell [6]. The blisters are unsightly and, if punctured, can release sulphurous smelling material [7]. *Polydora* sp. may compete with oysters for food [8], and can retard oyster growth resulting in undersized shells and reduced meat weight [6]. A Portuguese oyster breeding program in Vietnam, using phenotypic selection has commenced to reduce the incidence of these diseases [9,10]. Morphometric traits (shell length, shell width, shell depth, shell weight), sensory traits (tenderness and taste, and moisture content) are of economic importance in the oyster breeding program [9,10]. The family-based selection methods use the information from sibs; therefore, this results in lower accuracy using direct information from selection candidates. Advances in genomic technologies have enabled the incorporation of genomic information into breeding programs, which has increased selection accuracy, especially for traits that are difficult or expensive to measure or have low heritability [11,12].

Genomic selection was first proposed by Hayes and Goddard [13] and is an effective way to predict the phenotypic performance of individuals using high-density genetic markers [14]. It can exploit within family variability more accurately than pedigree information and has been widely shown to be more efficient by improving predictive accuracy and increasing genetic gain relative to conventional selection methods [12]. To date, prediction accuracies have been reported in several fish and shrimp species, ranging from 0.16 to 0.83 for growth, carcass and meat quality traits, and disease resistance [15,16,17,18,19,20,21]. In molluscs, genomic selection is less common, but has been used to evaluate growth traits [22], disease resistance [23] in the Pacific oyster *Crassostrea gigas*, and pearl quality traits in the Pearl oyster *Pinctada maxima* [24]. Although genomic selection has been routinely adopted in other industries, it has not yet been widely used in aquaculture. The main reason for the limited adoption in aquaculture is probably the relatively high cost of whole genome sequencing and genotyping arrays, especially for non-model species. Genotyping by sequencing is a low-cost alternative to using SNP arrays. Among the Restriction-site Associated DNA sequencing (RAD-seq) methods, DArTseq^TM^ is a combination of a DArT complexity reduction and next generation sequencing [25]. DArTseq^TM^ can be optimized for each species and application by selecting the most suitable complexity reduction method to accommodate the size and fraction of the genome selected for assay, and can generate data for a large number of markers. The cost per sample can be as low as $20–35 USD depending on marker density. An understanding of the relationship between genotyping costs and genomic prediction accuracy is necessary to find an appropriate balance between the two that will allow commercial adoption of genomic selection in the oyster breeding program. DArT-Seq has been used for genomic selection in other aquaculture species where a whole genome sequence is not available such as the banana shrimp [16] and yellowtail kingfish [15]. However, no studies to date have reported the use of DArT-Seq to evaluate prediction accuracy of genomic selection in a mollusc species.

The main aim of this study is to report on the application of genomic selection for genetic improvement of nine important traits that affect economic values and customers’ preferences, namely shell length, shell depth, shell width, shell weight, taste, and tenderness, moisture content, and *Polydora* sp. and *M. chungmuensis* prevalence. DArT-Seq was used to obtain genome-wide SNPs in commercially bred Portuguese oysters, which were then used to estimate the genetic parameters for the above traits and their prediction accuracy under several selection scenarios.

## 2. Material and Methods

### 2.1. Oyster Provenance

The Portuguese oyster used in this study were obtained from an ongoing breeding program for improved growth rate and the management of the oyster population was previously described in Vu et al. [9,26]. The oyster samples were collected and traits of interest recorded at harvest after nine months of culture.

### 2.2. Phenotypic Measurements

A total of 647 samples were collected, consisting of 188 oysters representing 57 full-sib families from the first generation and 459 oysters representing 33 full-sib families from the second generation in the Portuguese oyster breeding program at nine months old when oysters reached a marketable size [9]. The number per family ranged between 2–8 and 12–15 oysters for the first and second generation, respectively. All tissue samples were preserved in 80% ethanol and kept frozen at −80 °C until required. The population structure of individual samples is shown in the Supplementary Figure S1.

Shell length, shell width and shell depth were measured with calipers and an electronic scale was used for shell weight and wet weight of the entire oyster. Tenderness was assessed by pushing one finger on meat muscle and recorded as 1 for soft meat and 0 for firm meat. Taste was evaluated by the sensory testing panel to determine whether the soft tissue tasted salty (1) or mild (0) [10]. The soft tissue of the oyster was blotted dry with adsorbent paper and wet weight was determined with an electronic scale to an accuracy of 0.01 g. The tissue was then oven dried at 130 °C for 2 h and moisture content was calculated from the reduction in weight after drying. Polychaete infestation (*Polydora* sp.) was assessed by the presence of dark spots (marks, blisters) on the inner valves of the oysters [27]. The presence of *M. chungmuensis*, was inferred from spots (marks) present on the oyster gonad at harvest. Disease traits were recorded as either 1 for the presence or 0 for the absence of the particular parasite. Supplementary Figure S2 and Figure S3 show clinical signs of these parasite diseases on tissue and shell, respectively. A detailed procedure on how to record the edibility and disease related traits is given in Vu et al. [10].

### 2.3. DNA Extraction, Library Construction, SNP Genotyping and Quality Control

Genomic DNA was extracted and purified by Diversity Array Technology Pty Limited (Supplementary Table S1) with a DNA concentration of 2 µL per sample. DArTseq™ represents a combination of a DArT complexity reduction methods and next generation sequencing platforms [25,28,29]. Therefore, DArTseq™ represents a new implementation of sequencing of complexity reduced representations [30] and more recent applications of this concept on the next generation sequencing platforms [31,32]. Similarly, to DArT methods based on array hybridisations the technology is optimized for each organism and application by selecting the most appropriate complexity reduction method (both the size of the representation and the fraction of a genome selected for assays). Based on testing several enzyme combinations for complexity reduction Diversity Arrays Technology Pty Ltd. (Canberra, Australia) selected the PstI-SphI method for the Portuguese oyster. DNA samples were processed in digestion/ligation reactions principally as per Kilian et al. [25] but replacing a single PstI-compatible adaptor with two different adaptors corresponding to two different restriction enzyme (RE) overhangs. The PstI-compatible adapter was designed to include Illumina flowcell attachment sequence, sequencing primer sequence and “staggered”, varying length barcode region, similar to the sequence reported by Elshire et al. [32]. Reverse adapter contained flowcell attachment region and SphI-compatible overhang sequence. Only “mixed fragments” (PstI-SphI) were effectively amplified in 30 rounds of PCR using the following reaction conditions: an initial denaturation for 1 min at 94 °C, followed by 30 cycles of 94 °C for 20 s, 58 °C for 30 s, and 72 °C for 45 s; with a final extension at 72 °C for 7 min.

After PCR equimolar amounts of amplification products from each sample of the 96-well microtiter plate were bulked and applied to c-Bot (Illumina) bridge PCR followed by sequencing on Hiseq2500 (Illumina, San Diego, CA, USA). The sequencing (single read) was run for 77 cycles. Sequences generated from each lane were processed using proprietary DArT analytical pipelines. In the primary pipeline the fastq files were first processed to filter away poor quality sequences, applying more stringent selection criteria to the barcode region compared to the rest of the sequence. In that way the assignments of the sequences to specific samples carried in the “barcode split” step were very reliable. Filtering was performed on the raw sequences using the following parameters: filter with filter parameters, barcode region with Min Phred pass score 30 and Min pass percentage 75, whole read with Min Phred pass score 10 and Min pass percentage 50.

Approximately 2,500,000 sequences per barcode/sample were identified and used in marker calling. Finally, identical sequences were collapsed into “fastqcoll files”, which were “groomed” using DArT PL’s proprietary algorithm to correct for low quality bases. The “groomed” fastqcoll files were used in the secondary pipeline for DArT PL’s proprietary SNP and SilicoDArT (presence/absence of restriction fragments in representation) calling analysis algorithms DArTsoft14, which clustered all tags from all libraries using DArT PL’s C++ algorithm at the threshold distance of 3 for SNP calling. Technical parameters, especially the balance of read counts for the allelic pairs, were used to parse into separate SNP loci. Additional selection criteria included analysis of approximately 1000 controlled cross populations. Testing for Mendelian distribution of alleles in these populations facilitated selection of technical parameters discriminating well true allelic variants from paralogous sequences. In addition, multiple samples were processed from DNA to allelic calls as technical replicates and scoring consistency was used as the main selection criteria for high quality/low error rate markers. Calling quality was assured by high average read depth per locus (average across all markers was over 30 reads/locus). A total of 18,849 SNPs were produced after quality control under DArTseq standard, and 11,766 SNPs passed quality filtering with call rates of 50% for markers and samples that used for the downstream analyses.

### 2.4. Genetic Parameter Estimation

Genetic parameters were estimated using a genomic best linear unbiased prediction (GBLUP) approach. Univariate linear mixed models (model 1, below) were used to estimate the variance components and trait heritabilities. A total of 11,492 SNPs was used to estimate genetic parameters in this Portuguese oyster population:

(1) y=Xb+Za+e (Model 1)

where y is the vector of phenotypic measurements, b is the fixed effects vector (generation, sex), a is the vector of random genetic effects, e is the vector of residual errors, and X and Z are the design matrices for the fixed and random effects. The distributional assumption of the random effects was multivariate normal with mean zero and var $\left[\begin{array}{c} a\\ e \end{array}\right]$ = $\left[\begin{array}{cc} {G\sigma }_{\alpha }^{2} & 0\\ 0 & {I\sigma }_{e}^{2} \end{array}\right]$, where $\sigma_{\alpha }^{2}$, and $\sigma_{e}^{2}$ are additive genetic, and residual variance, respectively. G is the genomic relationship matrix obtained from the SNP markers and I is an identity matrix. Heritabilities were calculated as the ratio of $\sigma_{\alpha }^{2}$ and $\sigma_{p}^{2}$, where $\sigma_{p}^{2}$ is the phenotypic variance and calculated as $\sigma_{p}^{2}$ = $\sigma_{\alpha }^{2}$ + $\sigma_{e}^{2}$. Heritability estimates for binary traits (tenderness, taste, *Polydora* sp. and *Marteilioides chungmuensis*) were analysed on the observed binary scale [33].

Genetic correlations between traits using SNP marker information were estimated with bivariate models. The bivariate models were an extension of the univariate models as described in Mrode [34]. The fixed and random effects in the bivariate models were the same as in the univariate models. All these analyses were conducted with the ASReml 4.2 software [35].

### 2.5. Genomic Prediction Accuracy

Our study evaluated 3 methods for genomic prediction: BayesA [13], BayesCπ [36], and GBLUP [37]. In the GBLUP method, the variance of each SNP effect is assumed equal in the prior distribution [13,33]. The BayesA approach assumes a t distribution for marker effects and BayesCπ assumes that only a small fraction of the SNPs have non-zero effects [13,36,38]. 9942 SNP markers with a minor allele frequency > 0.05 were used for this evaluation including the first and second generations. The model 1 was used for GBLUP evaluation and model 2 was used for the Bayesian methods. The statistical model is: *y* = *Xb* + $\Sigma_{j=1}^{a}$*A_ij_g_j_ + e* (Model 2); where *y* is the phenotypic vector; *Xb* is the fixed term, where *b* is a vector containing fixed effects: generations, sex, and $\Sigma_{j=1}^{a}$*A_ij_g_j_* is the estimated genomic breeding value (GEBV) of individual *i* with *A* is the marker matrix and *g* are the marker effects. All effects of markers, phenotypic and residual variance, and GEBVs estimated by BayesA and BayesCπ were carried out using the R package BGLR [39]. The MCMC Gibbs sampling chain was run for 15,000 iterations, and the first 3000 iterations were discarded as burn in. For the GBLUP method, all variance components and GEBVs were analyzed using ASReml 4.2 [35].

### 2.6. Cross Validation

Five-fold cross-validation was used to evaluate the prediction accuracy (training set 80% and validation set 20%). Phenotypes from the validation population were masked and predicted from the training population. Prediction accuracy was calculated as the correlation between the estimated breeding values (EBVs) of the validation set and the actual phenotypes divided by the square root of the estimated heritability of total population. Mean prediction accuracy values obtained from the different sets were computed and compared between pedigree and genomic approaches.

Two strategies to assess the effects of marker density on the accuracies of genomic prediction were used gBLUP method. First, the density of the SNP panel used to build the genomic relationship matrix was changed by progressively increasing the minor allele frequency (MAF) threshold from 0.01 (almost all SNPs) to 0.475 (very few SNPs with balanced allele frequencies), resulting in progressively fewer markers being used. For the second strategy, the SNP panel members were randomly selected from the full dataset with three replicates for each panel size.

## 3. Results and Discussion

### 3.1. Trait Summary and Genetic Parameter Estimates

The mean and standard errors for morphometric traits were: 7.42 ± 0.05 (cm) for shell length, 4.16 ± 0.03 (cm) for shell width, 2.76 ± 0.02 (cm) for shell depth, 38.60 ± 0.60 (g) for shell weight. Those for edibility traits were 12.55 ± 0.28 (%) for moisture content, 0.05 ± 0.01 for taste, 0.91 ± 0.02 for tenderness. For disease traits they were: 0.26 ± 0.02 for *Polydora* sp. and 0.13 ± 0.02 for *M. chungmuensis* (Supplementary Table S2). Heritability estimates for morphometric traits ranged between moderate and high (h^2^ = 0.28–0.72) and not unexpectedly, were substantially higher than those obtained for disease related traits, which were 0.11 for *Polydora* sp. prevalence and 0.10 for *M. chungmuensis* (Table 1). Our estimates of genomic heritability for morphometric traits (h^2^ = 0.50–0.55) were considerably higher than those reported for the Pacific oyster, *Crassostrea gigas* (h^2^ = 0.23–0.26) [22] but were similar to those reported for the Zhikong scallop, *Chlamys farreri* (h^2^ = 0.39–0.54) [40]. The differences on heritability estimates among these studies can come from the culture method, biological characteristics of species or selection methods. These results suggest that genetic variance in the morphometric traits was abundant, and there is considerable potential to improve these traits via genomic selection. However, there are no reports on heritability estimates for edibility traits using genomic information that can be compared with those obtained from this study. The high genomic estimates of heritability are encouraging for the adoption of genomic selection to improve edibility traits, which cannot be measured directly on selection candidates but can be used to predict breeding values based on the eating quality of their siblings through genomic relationship. Genomic estimates of heritability for edibility traits were significantly higher than estimates of heritability based on the pedigree in this population [10]. This result also confirms that genomic information can be extremely useful for traits that are difficult to measure.

Finally, the additive variance component estimates in disease related traits were small, with heritability estimates of 0.11 for *Polydora* sp. prevalence and 0.10 for *M. chungmuensis*, suggesting that there is limited scope for selection against these disease traits in this population using genomic information. Our heritability estimates for disease related traits were significantly lower than those obtained for *Ostreid herpesvirus* disease trait in the Pacific oyster [23]. However, the results from our study originated from a field trial with natural pathogen challenge compared with pathogen specific challenge in the Pacific oyster [23]. Interestingly, few studies have used genomic information to estimate genetic parameters for disease resistance traits in oysters but there have been numerous breeding programs using traditional selection approach such as: summer mortality in the Pacific oyster, *Crassostrea gigas* [41]; summer mortality/OsHV-1 detected in *C. gigas* [42]; *Bonamia ostreae* in flat oysters, *Ostrea edulis* [43]; *Marteilia sydneyi* in Sydney rock oyster, *Saccostrea glomerata* [44]; *B. roughleyi* and *M. sydneyi* in *S. glomerata* [44]; and *H. nelsoni* and *Perkinsus marinus* in the eastern oyster, *Crassostrea virginica* [45]. In general, heritability estimates using the genomic relationship were higher than those reported based on a pedigree-based relationship matrix.

In addition to genomic heritability, the genetic correlations estimated using genomic information among morphometric traits were generally moderate to high (0.50–0.90), suggesting that these traits can co-select via selective breeding. Selection for one trait will result in desirable changes in the remaining traits. The high genetic correlation indicates that pleiotropic effects, or physical linkage and linkage disequilibrium could exist [46], and a similar set of genes may be involved in expression of these traits [47]. These results were in line with those reported in the Pacific oyster using genomic or pedigree relationship [23]. In contrast, in this study, the genetic correlations between morphometric traits (shell length, shell width, shell depth) and edibility and disease traits were small or non-significant. These correlations agreed with those estimated using the pedigree relationship information from this population [10]. This suggests that selection for any of the morphometric traits (shell length, shell width, shell depth, shell weight) may not lead to substantial changes in edibility and disease traits. More importantly, the non-significant genetic correlations between whole weight and sensory traits suggest that selection for whole weight will not result in any undesirable changes to sensory traits. However, there were unfavourable genetic correlations between whole weight and parasite disease traits (*r_g_* = 0.35–0.37) that were in line with those previously reported in this population using a pedigree relationship matrix [10]. This can explain because oysters with fast growth may have fewer resources invested in defense or thinner shells can allow for *Polydora* infestation. Lastly, the favourably positive and significant genetic correlation between whole weight and moisture content (*r_g_* = 0.60) suggests that whole weight is driven by water not viscera in this Portuguese oyster population. All phenotypic correlations among these traits had slightly smaller magnitudes than the genetic correlations using genomic information (Table 1).

### 3.2. Reliability of Different Genomic Selection Methods

Table 2 shows the prediction accuracies of the nine traits obtained from the three genomic prediction methods. The prediction accuracies were higher in morphometric and edibility traits (0.475–0.794) than in disease related traits (0.240–0.300). Out of the three methods, GBLUP gave higher prediction accuracies in six of the nine traits but the differences in predictive abilities between the methods were not large, in agreement with previous studies [15,16,17]. Therefore, the GBLUP method, which is less computationally demanding than the other methods, was used to estimate genetic parameters such as heritability and genetic correlations for the traits studied here. Capturing the genetic relationship among individuals in the pedigree is more important than capturing linkage disequilibrium. It is also more convenient for industry deployment as it is calculated in a single step that allows the use of both genomic and pedigree information simultaneously [48,49].

### 3.3. Prediction Performance with Different Number of SNP Sets

**Figure 1:**
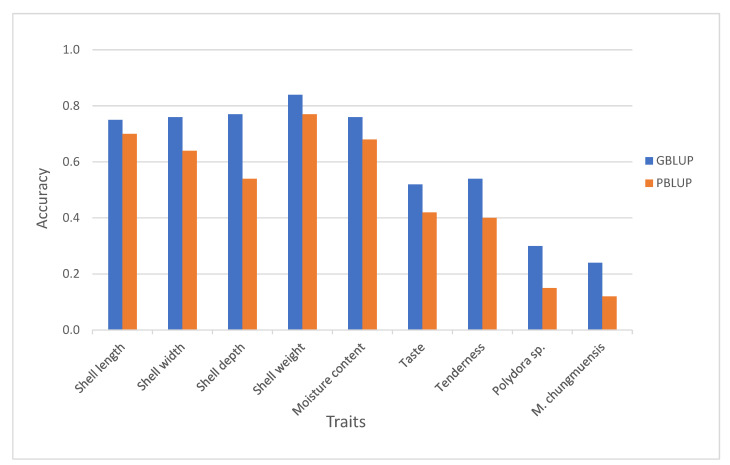
Predictive accuracies of GBLUP (Genomic Best Linear Unbiased Prediction) and PBLUP (Pedigree Best Linear Unbiased Prediction) for nine traits studied.

The prediction accuracy using the genomic information (G-matrix) for the nine traits studied were higher than that obtained using the Best Linear Unbiased Prediction using pedigree information (A matrix, PBLUP) (Figure 1, Table 3), with an increase of 15% in shell length (0.65 PBLUP to 0.75 GBLUP) to 200% for *Marteilioides chungmuensis* (0.12 PBLUP to 0.24 GBLUP) and for *Polydora* sp. (0.15 PBLUP to 0.30 GBLUP). Our study is in agreement with previous reports that indicate breeding value prediction accuracy to be higher using genomic information than pedigree relationships in aquaculture species [15,16,22,23,50,51,52,53,54,55,56]. The results of this study highlight the potential of genomic selection for economic traits in oysters. However, the genotyping cost remains a challenge for most aquaculture selective breeding programs. Therefore, a suitable strategy for covering genotyping costs must be devised to enable a wider adoption of genomic selection in the aquaculture industry.

**Figure 2:**
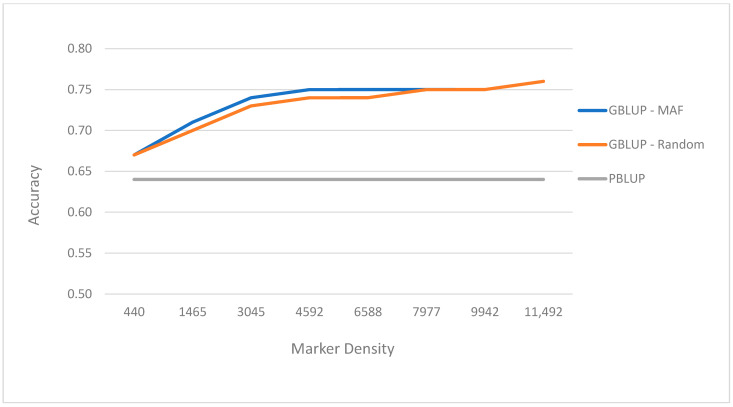
Prediction accuracy for shell width (cm) using GBLUP (Genomic Best Linear Unbiased Predictor) and MAF (minor allele frequency) and random approach and GBLUP at different marker densities as well as PBLUP (Pedigree Best Linear Unbiased Predictor).

To find a cost-effective genotyping strategy, we investigated the effects of SNP density on prediction accuracy of breeding values. The first series used subsets of SNPs defined by increasing the minor allele frequency (MAF) threshold leading to a decrease of SNP panel size. The second series involved randomly selecting the given number of SNPs to include in the panel with three replicates for each panel. The number of SNP markers had little impact on prediction accuracy until marker densities decreased below about 3000 SNPs. With the MAF approach, the genomic prediction accuracies obtained from lower density SNP panels ranged from 0.75 (MAF >0.05; 9942 SNPs) to 0.67 (MAF > 0.49; 440 SNPs) (Table 3 and Figure 2). Using the same numbers of SNPs but chosen randomly, there was no significant change in predictive accuracy relative to selecting the same number of SNPs using the MAF criterion. This suggests that there is large potential for using low-density SNP markers for genomic selection, which will be more affordable for aquaculture species. The prediction accuracies of genomic selection can be further improved by increasing the number of genotyped individuals from a more diverse population. This study was quite small involving only 188 broodstock in the first generation and 459 individuals in the second generation of the Portuguese oyster breeding program, and being a mix of full and half sib families, had potentially high levels of linkage disequilibrium across large chromosome segments. However, it is representative of typical aquaculture breeding schemes that utilize large full-sib families for sib testing and where genomic selection estimates with low-density markers can give high prediction accuracy [52]. A major impediment to widely using genomic selection in the aquaculture industry relates to the cost of genotyping and phenotyping selection candidates. The former can be tackled by using low-density SNP panels and our study suggests that low-density SNP panels may be adequate to achieve the accuracy needed for genomic selection in a mixed family oyster production system with shallow pedigrees. An alternative for a production system with a more diverse population is to use a mix of low- and high-density panels, where the broodstock is sequenced at a higher marker density and the offspring are sequenced at a lower density and then the lower density panels are imputed up to the higher density for the genomic prediction [57,58].

## 4. Conclusions

The moderate to high estimates of genomic heritability for edibility and growth traits; 0.44 for moisture content, 0.59 for taste, 0.72 for tenderness, and from 0.28 to 0.55 for shell length, shell width, shell depth and shell weight. Whereas, the genomic heritabilities were relatively low for the disease traits: *Polydora* sp. prevalence (0.11) and *M. chungmuensis* (0.10). Moderate to high and positive genetic correlations among whole weight and other morphometric traits (0.58–0.90); meanwhile, unfavourably positive genetic correlations were obtained between whole weight and the disease traits (0.35–0.37). There are no significant differences in prediction accuracy among three methods (GBLUP, BayesA, BayesCπ). Finally, there is large potential for using low-density SNP markers for genomic selection in this population.

## Figures and Tables

**Table 1:** Estimates (se) of heritabilities (on diagonal in bold), genetic correlations (below of diagonal), phenotypic correlations (above of diagonal) for whole weight (g), shell length (cm), shell width (cm), shell depth (cm), shell weight (g), moisture content (%), taste, tenderness, *Polydora* sp. and *Marteilioides chungmuensis* of the Portuguese oyster.

Traits	Whole Weight (g)	Shell Length (cm)	Shell Width (cm)	Shell Depth (cm)	Shell Weight (g)	Moisture Content (%)	Taste	Tenderness	*Polydora* sp.	*Marteilioides chungmuensis*
Whole weight (g)		0.51 (0.04)	0.47 (0.04)	0.51 (0.04)	0.89 (0.01)	0.58 (0.04)	n.e	n.e	0.34 (0.02)	0.27 (0.03)
Shell length (cm)	0.67 (0.09)	**0.50 (0.07)**	0.28 (0.05)	0.16 (0.06)	0.50 (0.04)	0.26 (0.05)	0.06 (0.03)	0.10 (0.05)	n.e	n.e
Shell width (cm)	0.58 (0.10)	0.58 (0.11)	**0.55 (0.06)**	0.34 (0.05)	0.45 (0.04)	0.26 (0.05)	0.13 (0.05)	0.02 (0.01)	n.e	n.e
Shell depth (cm)	0.63 (0.13)	0.50 (0.10)	0.84 (0.11)	**0.28 (0.08)**	0.48 (0.04)	0.16 (0.06)	n.e	n.e	n.e	n.e
Shell weight (g)	0.90 (0.03)	0.70 (0.08)	0.54 (0.10)	0.63 (0.12)	**0.42 (0.07)**	0.04 (0.01)	n.e	n.e	0.03 (0.01)	n.e
Moisture content (%)	0.60 (0.12)	0.11 (0.04)	0.36 (0.12)	0.07 (0.02)	0.06 (0.01)	**0.44 (0.07)**	0.12 (0.05)	n.e	n.e	n.e
Taste	n.e	0.07 (0.01)	0.19 (0.05)	n.e	n.e	0.14 (0.02)	**0.59 (0.06)**	0.07 (0.02)	0.02 (0.01)	n.e
Tenderness	n.e	0.12 (0.06)	0.06 (0.01)	n.e	n.e	0.07 (0.02)	0.15 (0.05)	**0.72 (0.06)**	n.e	n.e
*Polydora* sp.	0.37 (0.02)	n.e	n.e	n.e	0.04 (0.01)	n.e	0.07 (0.02)	n.e	**0.11 (0.04)**	0.23 (0.03)
*Marteilioides chungmuensis*	0.35 (0.05)	n.e	n.e	n.e	n.e	n.e	n.e	n.e	0.66 (0.12)	**0.10 (0.05)**

se: standard errors of mean; standard errors of genetic and phenotypic correlations in brackets; n.e: not estimable due to unloglikehood.

**Table 2:** Prediction accuracies (mean ± se) of BayesA, BayesCπ and GBLUP for whole weight (g), shell length (cm), shell width (cm), shell depth (cm), shell weight (g), moisture content (%), taste, tenderness, *Polydora* sp. and *Marteilioides chungmuensis* of the Portuguese oyster.

Methods	Traits								
	Shell Length (cm)	Shell Width (cm)	Shell Depth (cm)	Shell Weight (g)	Moisture Content (%)	Tenderness	Taste	*Polydora* sp.	*Marteilioides chungmuensis*
BayesA	0.734 ± 0.012	0.747 ± 0.101	0.730 ± 0.014	0.780 ± 0.023	0.475 ± 0.122	0.577 ± 0.025	0.485 ± 0.035	0.287 ± 0.016	0.242 ± 0.016
BayesCπ	0.732 ± 0.013	0.748 ± 0.103	0.695 ± 0.012	0.766 ± 0.025	0.523 ± 0.012	0.526 ± 0.021	0.481 ± 0.062	0.295 ± 0.021	0.241 ± 0.021
GBLUP	0.751 ± 0.024	0.750 ± 0.123	0.677 ± 0.015	0.794 ± 0.124	0.504 ± 0.020	0.599 ± 0.078	0.488 ± 0.065	0.300 ± 0.035	0.240 ± 0.035

GBLUP: Genomic best linear unbiased prediction.

**Table 3:** Genomic prediction accuracies for shell width using progressive increase the minor allele frequency (MAF) and random approach and PBLUP and BLUP methods.

Method	Approach	SNP	Accuracy	Approach	SNP	Accuracy
PBLUP	Pedigree	-	0.64	-	-	-
GBLUP	MAF 0.01	11,492	0.76	Random	11,492	0.76
GBLUP	MAF 0.05	9942	0.75	Random	9942	0.75
GBLUP	MAF 0.10	7977	0.75	Random	7977	0.75
GBLUP	MAF 0.15	6588	0.75	Random	6588	0.74
GBLUP	MAF 0.25	4592	0.75	Random	4592	0.74
GBLUP	MAF 0.35	3045	0.74	Random	3045	0.73
GBLUP	MAF 0.45	1465	0.71	Random	1465	0.70
GBLUP	MAF 0.49	440	0.67	Random	440	0.67

PBLUP: Pedigree best linear unbiased prediction; GBLUP: Genomic best linear unbiased prediction.

## Data Availability

The data have been used for the manuscript that are available from the corresponding author depending on reasonable request.

## Supplementary Materials

The following are available online at https://www.mdpi.com/2073-4425/12/2/210/s1, Figure S1: Principal component analysis of the relationship matrix for 647 oysters used in this study to show the population structure of individuals sampled, Figure S2: Oyster tissue was considered as an abnormal sign caused by *Marteilioides chungmuensis* parasites showing by arrows, Figure S3: Inner shell disease of oyster was caused by *Polydora* sp. showed by arrows, Table S1: DNA extraction procedure using Dart-seq technology, Table S2: Phenotypic measurements of the traits studied in this population.
