# Supplementary material for: Prediction Accuracies of Genomic Selection for Nine Commercially Important Traits in the Portuguese Oyster (Crassostrea angulata) Using DArT-Seq Technology
Source: Genes (Basel). 2021 Feb 1;12(2):210. doi: 10.3390/genes12020210 (PMC7910873; doi:10.3390/genes12020210)
Supplement: Supplementary file 1 [file genes-12-00210-s001.pdf]

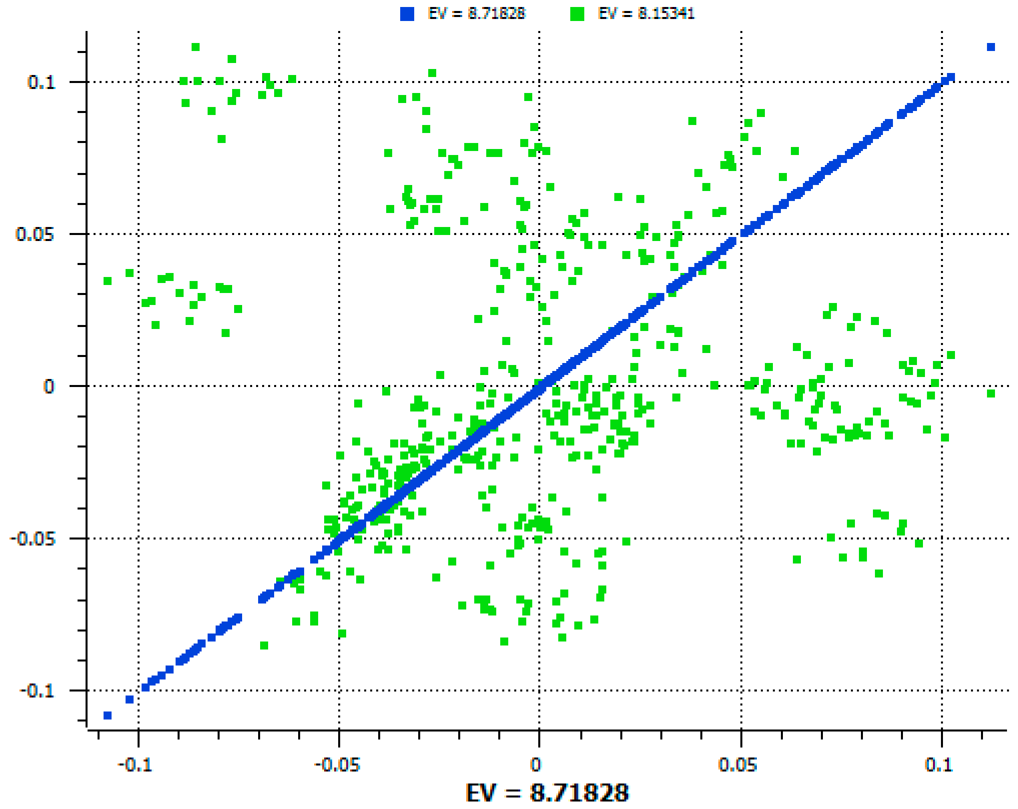

Supplementary Figure S1. Principal component analysis of the relationship matrix for 647 oysters used in this study to show the population structure of individuals sampled in this study

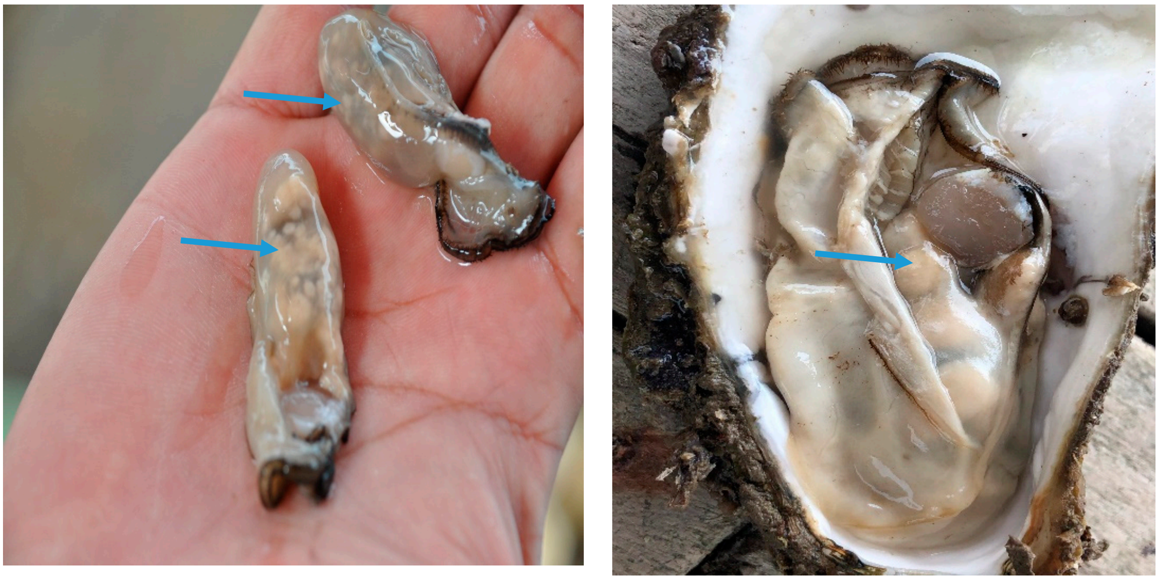

Supplementary Figure S2. Oyster tissue was considered as an abnormal sign caused by *Marteilioides chungmuensis* parasites showing by arrows

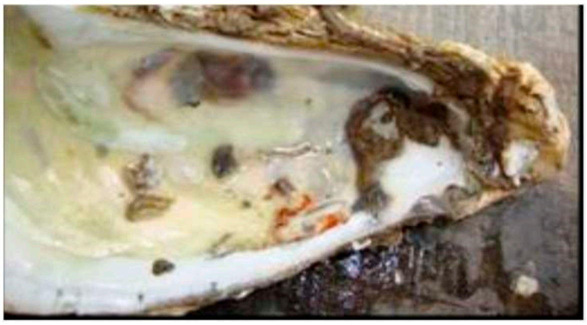

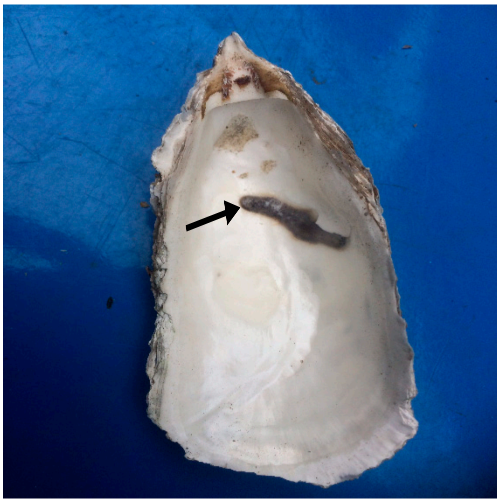

Supplementary Figure S3. Inner shell disease of oyster was caused by *Polydora* sp. showed by arrows

Supplementary Table S1. DNA extraction procedure using Dart-seq technology is as follows:

Procedure	Content
Step 1	Centrifuge plate at 2000 rpm for 3 minutes.
Step 2	Carefully unseal the columns of PCR plate.
Step 3	Pipette out all Ethanol using a multichannel pipette, and place the plate in the incubator at 60°C to allow all ethanol to evaporate.
Step 4	Lyse samples: For sample use 50µL of T1 Buffer and 6.25µL of proteinase K. Dispense 56.25µL of lysis solution into each sample, place the strip caps firmly and vigorously vortex the plate (use flat strip of 8 caps from Sarstedt to avoid leakage). Centrifuge plates briefly (30 to 60 sec at 1000 rpm) to collect any sample at the bottom of the wells. The tissue samples must be submerged in the solution. Incubate overnight at 60°C and to prevent any evaporation.
Step 5	Clear Lysate: Centrifuge for 10 min at 3000 rpm (program 5 on the centrifuge). Aspirate as much clear lysate as possible without touching any tissue leftover, transfer into the labelled deep well plate.
Step 6	Bind DNA to NucleoMag B-beads. Each sample need 6µL beads suspended in 90µL MB2. Measure Beads/MB2 volumes accurately and combine in a tube (15/50mL falcon tube/ 2mL Eppendorf tube for few samples), place cap on and mix well to suspend beads. Pour premixed Beads/MB2 solution into a reservoir, dispense 96µL to each sample. Ensure to gently agitate the reservoir at all times whilst dispensing to prevent the beads from settling down. Transfer the deep well plate to T100 robot.
Step 7	Final extraction steps (washing and elution into 30 uls of Elution Buffer) is done on Tecan 100 robot using 96 tips head and DArT PL script.

Supplementary Table S2. Phenotypic measurements of the traits studied in this population

Traits	Shell length (cm)	Shell width (cm)	Shell depth (cm)	Shell weight (g)	Moisture content (%)	Taste	Tenderness	*Polydora* sp.	*Marteilioides chungmuensis*
Mean (se)	7.42 (0.05)	4.16 (0.03)	2.76 (0.02)	38.60 (0.60)	12.55 (0.28)	0.05 (0.01)	0.91 (0.02)	0.26 (0.02)	0.13 (0.02)

se is the standard error
